# The percent of chronic migraine patients who responded to onabotulinumtoxinA treatment per treatment cycle in the PREEMPT clinical program

**DOI:** 10.1186/1129-2377-14-S1-P200

**Published:** 2013-02-21

**Authors:** S Silberstein, DW Dodick, RE DeGryse, R Lipton, CC Turkel

**Affiliations:** 1 Thomas Jefferson University, Philadelphia, PA, USA; 2 Mayo Clinic Arizona, Phoenix, AZ, USA; 3 Allergan, Irvine, CA, USA; 4 Albert Einstein College of Medicine, Bronx, NY, USA

## Introduction

CM is a prevalent, disabling neurological disorder. OnabotulinumtoxinA is the only approved therapy specifically for CM. In patients who do not respond to the first onabotulinumtoxinA injection cycle, it is unclear whether subsequent injections cycles will be effective.

## Objective

To determine the proportion of first-time responders with chronic migraine (CM) who demonstrate a clinically meaningful response to onabotulinumtoxinA in the first 3 treatment cycles of the PREEMPT clinical program.

## Design/methods

PREEMPT (two phase 3 studies: 24-week, double-blind, placebo-controlled, parallel-group phase, followed by 32-week, open-label phase) evaluated onabotulinumtoxinA for prophylaxis of headaches in CM (≥15 days/month with headache lasting ≥4 hours/day). Patients were randomized (1:1) to onabotulinumtoxinA (155-195U) or placebo every 12 weeks. We evaluated 50% responder rate for three treatment cycles across multiple efficacy variables. This rate exceeds the previously suggested clinically meaningful response rate of 30% in patients with CM.1

## Results

Pooled analyses demonstrated high responder rates among onabotulinumtoxinA-treated patients (n=688) after Treatment Cycle 1 in frequency of headache days (49.3% of patients), moderate/severe headache days (53.0%), and cumulative hours of headache on headache days (54.2%) and a 21;5-point improvement in HIT-6 (56.3%). After Treatment Cycle 2, an additional 11.3-14.5% of patients who did not respond to Treatment Cycle 1 became responders. With a third treatment, an additional 7.4-10.3% of patients became responders.

## Conclusions/relevance

These data demonstrate that a high proportion of onabotulinumtoxinA-treated patients are responsive (50% improvement) to the first treatment cycle, and patients who were not responders to the first cycle may become responders with a second and/or third treatment cycle.

## Support

Allergan, Inc.
